# Pleuritis and Pneumo-Thorax

**Published:** 1831

**Authors:** 


### XLIII.

### PLEURITIS AND PNEUMO-THORAX.

THIS is a case related by Mr. Smeal, published in the first number of a new periodical (the Glasgow Medical Examiner) for April last. The patient was a young man, a cotton-spinner, who became affected in March, 1829, with cough and hæmoptysis, after exposure to cold. For these he took no advice, but went to Ireland, where his health was much improved. In January 1830, the hæmoptysis returned in considerable quantity, and again ceased without medicine. In the Spring of the same year he was seized with acute pain in his left side, which confined him for seven weeks to bed. The febrile symptoms subsided, but left a dry cough, and some dyspnœa. When first seen by Mr. Smeal, 28th April, 1830, he was debilitated, emaciated, and worn down with dry cough and difficulty of breathing.

"The patient being stripped, the epigastric and hypochondriac regions appear more prominent and feel more tense than natural; but no other symptoms of disease of the viscera of these parts. The action of the heart is felt and seen under the cartilages in the right side of the chest, instead of in the left; left side of chest is visibly larger than the right, both when standing on a level with the patient and on a chair behind him looking down upon him, but careful measurement shows little or no difference; left intercostal spaces tense, but no sensible fluctuation. *Immediate Percussion*—Complete mattité of sound in the left side of chest, except in the space of three fingers' breadth below the clavicle, in which space the sound is clear, but cannot be recognized to be more clear than same space in right side; right side of chest sufficiently sonorous, except the space there unnaturally occupied by the heart. *Mediate auscultation*—Total absence of the respiration in the left side, including the clear sounding space below the clavicle; except at the root of the lung over the large bronchial tubes in the space between the basis of the scapula and spine, where the bronchial respiration is heard very distinctly, clear, sonorous, and without any mixture of rale, egophony could no where be detected. In the right side of the chest, except in the space occupied by the heart, the respiration (both vesicular and bronchial), is very clear, strongly puerile, and without any mixture of rale.—Stethoscopic examination of the heart in its unnatural situation, indicates a perfectly healthy state of its walls, cavities, and valves.

From these negative and positive symptoms, no doubt could be entertained of extensive pleuritic effusion in the left side of the chest, compressing the lung into a small space against the spine, totally preventing the entrance of air into its vesicles and smaller bronchial tubes, and pressing the heart and mediastinum a considerable distance into the right side of the chest; whilst the absence of the respiration, and the clearness of sound in the space of three fingers' breadth below the clavicle, clearly indicated a slight complication of pneumo-thorax: At the same time, the absence of characteristic expectoration, of tintement metallique, of bourdonnement amphorique, and of souffle of every kind, showed that, at present at least, there was no communication between the effused fluid and the bronchiæ."

Paracentesis thoracis appeared to Mr. Smeal as the only remedial measure that promised a cure or even relief; but as this was a serious operation in its results, he first tried some active means of producing absorption. Purgatives, diuretics, &c. were employed, but without any good effect. On the 29th of May, paracentesis, between the fourth and fifth ribs, was performed, by means of a large-sized hydrocele trocar, when 21 gills, imperial measure, of a transparent yellow fluid were evacuated. June 3d. The patient is much relieved in all respects, and can now lie on either side—pulse 108—heart's action still visible in the right side, but nearer the sternum than before the operation. Left side of chest very sonorous, but no respiration to be heard. Large blister to that side—purgatives. 26th June. The patient is now nearly in the same state as before the paracentesis. The operation was repeated, and 18 gills of similar fluid abstracted. The wound was enlarged by a probe-pointed bistoury, so as to admit the point of the fore-finger, and a tent was introduced, with the intention of rendering it fistulous. In two or three days the tent was withdrawn, and another introduced, as there were no symptoms of inflammation or fever. On the fifth day, however, we find him with fever, bilious vomiting, heat of chest, inflammation of pleura, and emphysema of left side of chest, face, and arm, produced by the air forced out of the chest by coughing. A third tent was introduced. Calomel and rhubarb. 1st July, Inflammatory symptoms continue—canula introduced, and five ounces of *turbid* serum evacuated. The tent was not re-introduced; but the wound covered with adhesive plaister. 2d July. The canula again put in, seven ounces of decidedly *purulent* fluid extracted. Between the 5th and 16th July, the patient was visited every other day, and the canula introduced, followed by an average discharge of 16 ounces of purulent matter. The febrile symptoms gradually subsided, and the cough abated. The left lung is the same as before the operation. The heart now pulsates under the sternum. 30th July to 15th August, visited and treated as before.

The discharge is diminished to 12 ounces every second day. The patient is much improved in all respects. 7th Sept. Continues to improve. The discharge is now of an amber colour, and greatly diminished in quantity. From this time, the patient called at Mr. Smeal's house every fourth day to have the canula introduced.

"27th Sept. Continues to improve; coughs occasionally; expectoration the same as when he applied to me about five months ago; no vomiting; bowels and urine natural. Heart in its natural situation; pulse 96, of tolerable strength, respiration 25 times in a minute; in the right lung it is very energetic and decidedly puerile, being unusually clear and full, even as low as the 10th and 11th ribs, where it is generally very indistinct in most people. Left side of chest very much contracted; its antero-posterior measurement 5¼ inches, whilst that of the right is 8½ inches—difference 3¼ inches. The patient appears to lean to the left side in consequence of the shoulder being drawn downwards; the ribs at their curvatures are nearly in contact, as it is now difficult to introduce the canula; whilst the intercostal spaces towards the sternum, especially the lower ones, are very much depressed. A bougie introduced into the wound comes in contact with the diaphragm at the distance of 1¼ inch, with the heart and pericardium at 4½ inches, and with the lung compressed against the spine at 9¼ inches. From what is now related, it is pretty clear that all, or at least nine-tenths, of the blood sent out by the right ventricle is conveyed to the right lung only through the right division of the pulmonary artery; and that the left auricle must be supplied with the changed blood only by the right pulmonic veins, so that the right lung now performs double duty. Patient now called on me once a week—tonic treatment continued.

18th of October.—Continues better; ten days since last introduction of canula; discharge from chest at this time drawn off, being put into a graduated glass measure, amounts exactly to 6 ozs.; it is very tenacious and ropy, and exactly resembles the morbid discharge passed in the urine of persons affected with chronic inflammation of the mucous coat of the bladder. Having laid him down on his right side, I injected from 16 to 20 ozs. of tepid water into the left side of his chest, in order to try if coughing would cause any of it to come out by his mouth, but nothing of the kind took place; it flowed out again by the canula, when he turned to his left side, and I am convinced from all that has occurred, that since I first saw him there has been no communication between the bronchiæ and the pleura; but whether his case has been simple pleurisy, or pleurisy excited by the rupture of a tubercle without bronchial communication, or with it, and that communication again cut off by the gradual compression of the lung, is at present impossible to determine. He tells me, that, notwithstanding his present betterness, he is quite unfit for work, as any exertion excites very troublesome quickness and shortness of breathing, with a sensation as if his throat would burst, great fluttering at his heart and giddiness in his head.—Treatment as before."

This poor fellow, who deserved a better fate, was caught in a heavy shower, and wetted to the skin, early in November. Fever was lighted up—the discharge became purulent—and he was carried to his grave by hectic fever, on the 27th January of the present year. We shall give the post-mortem examination in the words of the author.

"28th.—*Sectio Cadaveris.* Messrs. J. Anderson and J. P. Glen, surgeons, were present. They had seen the patient since he came under my care, both before, at the time, and after the operation of paracentesis. The right side of the chest contained about ten ozs. of transparent yellow serum. The costal portion of the pleura healthy; the diaphragmatic and pulmonary portions intensely inflamed, and coated with recent false membrane as thick as writing-paper, which can be easily separated in small portions; the interlobular pleura is similarly affected, whereby the lobes are glued together. The lung is compressed towards the spine, into about the sixth part of its usual size, with its upper lobe adhering to the chest by short and firm membranous bands. The trachea having been divided, the pipe of a pair of bellows was introduced, and the trachea secured on it by a ligature, in order to attempt to inflate the lung, and, if possible, to discover the false opening; in the latter we were unsuccessful, and in the former successful only in a slight degree, owing partly to the false membrane, but mostly to the bellows not being air-tight. Having filled the side of the chest with water, and keeping the lung immersed, a second attempt was made to discover the opening, expecting to be led to it by seeing bells of air rising on the water when the bellows were quickly and strongly worked, but in this we were also disappointed; even after taking out the lung, and separating its interlobular adhesions, no traces of a false opening could we discover. The middle and lower lobes were healthy, and contained no tubercles; in the upper lobe they were numerous, but very small; at its uppermost part there was a tubercular excavation, which would contain a small nut, and which was filled by a recent coagulum of blood, having a branch extended into the communicating bronchial tube. The outer wall of this excavation consisted only of the pleura, and it was here that the perforation existed; the coagulum in the excavation and bronchial communication, and the false membrane covering the perforation in the pleura, were the causes of our not discovering this perforation by the use of bellows.

Left side of the chest much contracted.—Every part of the pleura coated by firmly-organized false membrane, about an eighth of an inch in thickness, and having a flocculent secreting surface—about 12 ozs. of purulent matter in this side of the chest. The left lung not visible by reason of the false membrane which binds it down against the spine. On being cut out, it is found to be about five inches long, three inches broad, and little more than one inch thick, and totally deprived of air; its lower lobe contains no tubercles, but they are numerous and small in its upper lobe; and in this, as in the other side of the chest, there is near its upper part a rather larger tubercular excavation filled with dark-coloured pulpy matter; but there is a portion of condensed pulmonary tissue between this excavation and the pleura. On the middle of the outside of the upper lobe, there is a nipple-like projection about the size of a small pea, which one of us conjectured to have been a place where a tubercle had burst into the pleural cavity sometime ago; but on cutting into it, nothing like tubercular residuum could be recognised, therefore its nature is quite uncertain.

Pericardium, heart, and abdominal viscera perfectly healthy. Slight lateral curvature of the spine, with the convexity to the right side of the chest, and in all probability produced by the contraction of the left side."

We cannot but doubt the propriety of the surgical treatment in the above case. The enlargement of the opening, and the introduction of tents kindled up inflammation, and converted a surface which hitherto secreted a transparent fluid, into one which secreted pus. Surely there was little gained by such a conversion. We should have been more inclined, either to have left a very small tube in the wound, or introduced one every three or four days, as was practised with advantantage in the sequel. There is a great probability that, had it not been for the exposure to cold in November, this man might have recovered a certain degree of health. The case, therefore, is a very interesting one.

---

